# Schlafen 12 Modulation and Targeting in Acute Myeloid Leukemia

**DOI:** 10.1158/2767-9764.CRC-25-0283

**Published:** 2025-11-17

**Authors:** Jamie N. Guillen Magaña, Markella Zannikou, Aneta Baran, Sara Small, Michael Schieber, Matthew J. Schipma, Elizabeth T. Bartom, Masha Kocherginsky, Diana Saleiro, Elspeth M. Beauchamp, Frank Eckerdt, Leonidas C. Platanias

**Affiliations:** 1 Robert H. Lurie Comprehensive Cancer Center of Northwestern University, Chicago, Illinois.; 2 Division of Hematology-Oncology, Department of Medicine, Feinberg School of Medicine, Northwestern University, Chicago, Illinois.; 3 Department of Medicine, Jesse Brown Veterans Affairs Medical Center, Chicago, Illinois.; 4 Department of Biochemistry and Molecular Genetics, Feinberg School of Medicine, Northwestern University, Chicago, Illinois.; 5 Division of Biostatistics and Informatics, Department of Preventive Medicine, Feinberg School of Medicine, Northwestern University, Chicago, Illinois.

## Abstract

We examined the role of SLFN12, a member of the Schlafen (SLFN) family of interferon-regulated genes and proteins in leukemogenesis, and its potential as a therapeutic target in acute myeloid leukemia (AML). We explored the effects of velcrins, a class of small molecules able to modulate SLFN12 biological activity, on AML cells. Velcrin treatment of AML cells stabilized SLFN12 and promoted SLFN12 complex formation with phosphodiesterase 3A or phosphodiesterase 3B. Such effects were associated with growth-inhibitory and proapoptotic responses, as well as potent suppressive effects on leukemic cell growth. In addition, velcrin treatment suppressed clonogenic capacity of primitive leukemic progenitors and significantly extended survival in a mouse AML xenograft model. Taken together, these findings establish an important role of SLFN12 in leukemogenesis and raise the potential for the use of velcrins as a therapeutic strategy for AML.

**Significance:**

Our studies identify SLFN12 as a potential target in AML with important clinical–translational implications.

## Introduction

Acute myeloid leukemia (AML) is an aggressive type of leukemia, with high morbidity and mortality and a very low overall survival rate, especially for older patients (1). The treatment of AML depends on age, subtype, and genetic abnormalities (1–6), while certain biomarkers can help define therapy planning (6). In recent years, there has been expanded use of hypomethylating agents such as 5′-azacitidine (Aza) or decitabine in combination with the BCL2 inhibitor venetoclax (3–5). Also, depending on molecular findings, inhibitors of Fms-like tyrosine kinase 3 (FLT3) and other targeted therapies (1–6) are used. However, such approaches have not yielded major outcome improvements for the majority of patients with AML (1–3), underscoring the need for the identification of novel cellular targets for the design of new effective treatments and options.

Schlafens (SLFN) are interferon-inducible genes with emerging roles in the regulation of immune and antiviral responses (7–17). Human SLFN family members include SLFN5, SLFN11, SLFN12, SLFN12L, SLFN13, and SLFN14, of which SLFN5 and SLFN14 seem to represent mouse homologs (7). The function and regulation of members of this family of genes and proteins have recently been of high interest in the development of novel anticancer therapies (7, 10, 18–20). For instance, SLFN11 sensitizes malignant cells to DNA-targeted antineoplastic drugs (21, 22), whereas SLFN5 may serve as a biomarker in a variety of cancers (23). In addition, *SLFN12* mRNA levels are elevated in certain tumor types (20, 24, 25).

Despite the substantial interest toward the development of drugs modulating SLFN activity, no direct SLFN activators or inhibitors have been developed so far. Velcrins, a class of small molecules that act like molecular glues by inducing a heterotetrameric complex between SLFN12 and the protein phosphodiesterase 3A (PDE3A), can promote cancer cell death by stabilizing SLFN12 protein, thereby enhancing its RNase activity (26, 27). In fact, velcrin-based SLFN12 modulation has shown significant potential for treating certain SLFN12- and PDE3A-positive cancers (28). Anagrelide, zardaverine, and 6-(4-(diethylamino)-3-nitrophenyl)-5-methyl-4,5-dihydropyridazin-3(2H)one (DNMDP) are velcrins that have been extensively studied as antineoplastic agents (29, 30). However, the structural instability of DNMDP has been a restrictive factor for its clinical development (31). Based on promising preclinical studies using BAY 2666605, a compound with improved stability (31), a first-in-human clinical trial (NCT04809805) using this agent was performed in solid tumors (26, 28). Here, we provide evidence that the velcrin BAY 2666605 promotes potent antileukemic effects in *in vitro* and *in vivo* AML models via modulation of SLFN12 activity through complex formation with either PDE3A or phosphodiesterase 3B (PDE3B).

## Materials and Methods

### *SLFN12* mRNA expression in different cancer cell lines

The expression of *SLFN12* mRNA across various cancer types was analyzed by employing The Cancer Genome Atlas (TCGA) Pan-Cancer dataset via the University of California Santa Cruz Xena browser (https://xena.ucsc.edu/, RRID: SCR_018938; refs. 32, 33).

### Cell lines and reagents

HEL (ATCC, cat. #TIB-180, RRID: CVCL_0001) and U937 (ATCC, cat. #CRL-1593.2, RRID: CVCL_0007) cells were cultured in RPMI-1640 medium (Gibco), 10% FBS (MilliporeSigma), and antibiotics [0.1 mg/mL gentamicin (Sigma-Aldrich)]. SET-2 (DSMZ, cat. #ACC-608, RRID: CVCL_2187) cells were cultured in RPMI medium, 20% FBS, and antibiotics. K-562 (ATCC, cat. #CCL-243, RRID: CVCL_0004) and MV-4-11 (ATCC, cat. #CRL-9591, RRID: CVCL_0064) cells were cultured in Iscove's modified Dulbecco's medium (Thermo Fisher Scientific), 10% FBS, and antibiotics. KG-1 (ATCC, cat. #CCL-246, RRID: CVCL_0374) cells were cultured in Iscove's modified Dulbecco's medium, 20% FBS, and antibiotics. OCI-AML-5 (ATCC, cat. #ACC-247, RRID: CVCL_1620) cells were cultured in minimum essential medium ɑ (ɑ-MEM; Gibco), 20% FBS, GM-CSF [10 ng/mL; PeproTech Recombinant Human GM-CSF (Thermo Fisher Scientific)], and antibiotics. Cell lines were cryopreserved at early passages in liquid nitrogen and maintained in culture for a maximum of 10 to 15 passages. All cells were cultured under standard conditions at 37°C with 5% CO_2_ and routinely subjected to *Mycoplasma* contamination testing using the MycoAlert PLUS Mycoplasma Detection Kit (Lonza), as suggested by the manufacturer’s protocol. All cell lines underwent short tandem repeat analyses and were authenticated using the ATCC or DSMZ database biannually. BAY 2666605 was purchased from MedChemExpress. Aza was purchased from TargetMol. Detailed information on cell lines and reagents can be found in the key resources table (Supplementary Table S1).

### Cell lysis and immunoblotting

To assess basal protein levels of SLFN12, PDE3A, and PDE3B in leukemia cells, cell pellets were collected 24 hours after seeding. To assess SLFN12 stabilization *in vitro*, HEL cells were treated with vehicle (DMSO) or BAY 2666605 at the indicated concentrations and collected 4 or 8 hours after treatment. To assess the expression of apoptotic markers cleaved PARP and cleaved caspase-3, HEL cells or U937 cells were seeded in a six-well plate and treated with the indicated doses of BAY 2666605 or vehicle (DMSO) for 24 hours. All cell pellets were lysed in NP-40 lysis buffer [50 mmol/L Tris-HCL (pH = 7.5), 150 mmol/L NaCl, 0.2 mmol/L EDTA, and 0.5% NP-40]. To assess SLFN12 levels after BAY 2666605 treatment *in vivo*, tumors were homogenized using TissueRuptor II (Qiagen) and lysed using a 0.5% Triton lysis buffer (50 mmol/L HEPES, 120 mmol/L NaCl, 10 mmol/L Na pyrophosphate, 1 mmol/L EDTA, pH 8.0, 50 mmol/L NaF, 0.5% Triton X-100, and 10 mmol/L β-glycerophosphate). All lysates were freshly supplemented with 1 mmol/L phenylmethylsulfonylfluoride (Santa Cruz Biotechnology, cat. #sc-482875), 100 mmol/L of NaF (Sigma-Aldrich, cat. #S1504), 200 μmol/L Na_3_VO_4_ (New England Biolabs, cat. #P0758S), protease inhibitor cocktail set III (EMD Millipore, cat. #539134), and phosphatase inhibitor cocktail set I (EMD Millipore, cat. #524624). For all experiments, equal amounts of the protein lysate were resolved by SDS-PAGE and immobilized on an Immobilon-P polyvinylidene difluoride (Millipore) membrane using the Trans-Blot Turbo transfer system (Bio-Rad). Membranes were blocked with 5% BSA/milk in Tris Buffered Saline with Tween-20, followed by probing with primary and horseradish peroxidase (HRP)–conjugated secondary antibodies. The binding of antibodies was detected using Clarity Max ECL (Bio-Rad) or WesternSure PREMIUM Chemiluminescent Substrate (LICORbio) and visualized using the ChemiDoc MP imager (Bio-Rad), Odyssey XF (LICORbio), or via film. The following commercially available antibodies were used for immunoblotting analysis: SLFN12 rabbit monoclonal antibody (1:250–1:500, Abcam, cat. #AB234418, RRID: AB_3714881), PDE3A rabbit polyclonal antibody (1:1,000, Bethyl Laboratories, cat. #A302-740A, RRID: AB_10634214), PDE3B rabbit polyclonal antibody (1:1,000, Bethyl Laboratories, cat. #A302-743A, RRID: AB_10631431), GAPDH mouse monoclonal antibody (1:5,000, Thermo Fisher Scientific, cat. #4331182, RRID: AB_2107445), PARP polyclonal rabbit antibody (1:1,000, Cell Signaling Technology, cat. #9542S, RRID: AB_2160739), cleaved caspase-3 polyclonal rabbit antibody (1:500–1:1,000, Cell Signaling Technology, cat. #9661S, RRID: AB_2341188), anti–rabbit IgG HRP-linked antibody (1:2,000, Cell Signaling Technology, cat. #7074S, RRID: AB_2800208), anti–mouse IgG (H + L)-HRP conjugate (1:5,000, Bio-Rad, cat. #1706516, RRID: AB_11125547), and anti–rabbit IgG (light chain specific; D4W3E) mAb (HRP conjugate; 1:1,000, Cell Signaling Technology, cat. #93702S, RRID: AB_2800208). Immunoblots shown are representative of three independent experiments.

### siRNA-mediated inhibition of gene expression

The following ON-TARGETplus human siRNAs were purchased from Horizon Discovery (Dharmacon): non-targeting control pool siRNAs (D-001810-10-20) and SMARTpools targeting *SLFN12* (L-018142-02-0020), *PDE3A* (L-007645-00-0005), or *PDE3B* (L-007646-00-0005). HEL cells were transfected with siRNA targeting *SLFN12*, *PDE3A*, *PDE3B*, or a non-targeting control using the Lonza Cell Line Nucleofector Kit V (cat. #VCA-1003) and program X-005 per the manufacturer’s protocol. For experiments involving siSLFN12, cells were seeded 24 hours after transfection for WST-1 cell viability colorimetric assay (WST-1) or colony formation assay. Remaining cells were harvested for immunoblot or qRT-PCR analysis. For experiments involving siPDE3A/B, cells were treated 48 hours after transfection with BAY 2666605 or DMSO control at the indicated concentrations and collected at the indicated time points for subsequent qRT-PCR or immunoblot analysis.

### qRT-PCR

RNA isolation was conducted using the RNeasy Mini Kit (Qiagen, cat. #74106) per the manufacturer’s protocol. A total of 1 μg of mRNA was reverse transcribed into cDNA using the High-Capacity cDNA Reverse Transcription Kit (Thermo Fisher Scientific, cat. #4368814). qRT-PCR was performed using the Bio-Rad CFX96 Real-Time System machine (Bio-Rad) or the Applied Biosystems QuantStudio 6 Flex Real-Time PCR System (Thermo Fisher Scientific) using commercially available FAM-labeled probes and primers (Thermo Fisher Scientific) to determine the expression of human *SLFN12* (Hs01049939_m1), *PDE3A* (Hs01012698_m1), and *PDE3B* (Hs00265322_m1). Human *GAPDH* (Hs02758991_g1) was used for normalization. Relative quantitation of mRNA levels was calculated using the ΔΔCt method and graphed as percent change compared with control siRNA-transfected untreated cells.

### Co-immunoprecipitation assays

To study the physical interaction between PDE3A or PDE3B and SLFN12, cells were treated with either DMSO or BAY 2666605 with low (1 μmol/L) or high (10 μmol/L) concentrations of velcrin and collected 24 hours after treatment. To immunoprecipitate PDE3A or PDE3B, cell lysates were incubated overnight at 4°C with either anti-PDE3A rabbit polyclonal antibody (1:100), anti-PDE3B rabbit polyclonal antibody (1:100), or rabbit IgG polyclonal antibody isotype control (Proteintech, cat. #30000-0-AP, RRID: AB_2819035) with rotation. The next day, Protein G Sepharose 4 Fast Flow beads (GE Healthcare) were added for 1 hour at 4°C with rotation. Beads were then washed four times with NP-40 lysis buffer, and immunoprecipitated proteins were eluted using Lane Marker Reducing Sample Buffer (Pierce) at 95°C for 10 minutes. Samples were then resolved using SDS-PAGE, transferred to a polyvinylidene difluoride membrane, and subjected to immunoblotting.

### Drug synergy calculations

To determine whether drug interactions were additive, synergistic, or antagonistic, we calculated combination index (CI) values using Compusyn. CI values < 1 were considered synergistic, >1 were considered antagonistic, and = 1 were considered additive.

### Clonogenic leukemic progenitor assays in methylcellulose

To assess the effects of velcrins on leukemic progenitor [leukemic colony-forming unit (CFU-L)] colony formation, HEL or U937 cells were plated in a six-well dish in methylcellulose (MethoCult H4534 Classic without EPO, STEMCELL Technologies, cat. #04534) in the presence of BAY 2666605 at the indicated final concentrations. Cells were seeded and treated on the same day, and CFU-L was counted as in previous studies (22, 34). For studies with samples from patients with primary AML, peripheral blood or bone marrow samples were collected in heparinized tubes after obtaining written informed consent, and the study was approved by the Institutional Review Board of Northwestern University. Mononuclear cells were isolated by Ficoll-Hypaque (Sigma-Aldrich) gradient sedimentation and processed as described previously (22). Human leukemic progenitor cells (CFU-L) were assayed in MethoCult H4434 Classic medium (STEMCELL Technologies, cat. #04434) and assessed after 2 weeks (22).

### WST-1 cell viability assay

Cell viability was assessed by plating AML cell lines in triplicates in 96-well plates, as in previous studies (22, 34). Different lines were seeded at 1,500 to 2,000 cells per well in a 96-well plate. Cells were treated with vehicle control (DMSO), BAY 2666605 velcrin (BAY), Aza, or Aza in combination with BAY at the indicated concentrations. After 4 days, cell viability was measured using the WST-1 cell proliferation reagent following the manufacturer’s protocol (Roche). Absorbance was measured at 450 and 600 nm (reference wavelength) using an Epoch Plate reader and Gen5 software (BioTek Instruments Inc, RRID: SCR_017317). IC_50_ values were calculated using Prism GraphPad.

### Assessment of apoptosis by flow cytometry

Cells were seeded in a six-well plate and treated in a time- and dose-dependent manner as indicated. At the specified time points, cells were harvested by transferring them with complete medium into 1.5-mL reaction tubes. Cells were centrifuged at 500 × *g* for 5 minutes at 4°C, and the supernatant was aspirated. Cells were washed with 1 mL PBS and resuspended in 100 μL of staining buffer (FITC Annexin V Apoptosis Detection Kit I) by mixing 2 μL of annexin-V and 4′-6-diamidino-2-phenylindole (DAPI) in incubation buffer according to the manufacturer’s instructions (BD Biosciences). The cells were incubated for 15 minutes at room temperature and protected from light. After centrifugation at 500 × *g* for 5 minutes at 4°C, the staining buffer was aspirated, and cells were resuspended in 100 μL of PBS. Flow cytometric analyses were conducted immediately using an LSRFortessa flow cytometer (BD Biosciences), with cell gating and analyses performed using FlowJo software version 10.8.1 (Tree Star, RRID: SCR_008520).

### Animal studies

Animal experiment protocols were approved by the Northwestern University Institutional Animal Care and Use Committee and performed accordingly. Five- to six-week-old NU/NU nude female mice were acquired from Charles River Laboratories (strain code 088, RRID: IMSR_CRL:088). To induce flank tumors, 5 × 10^6^ early-passage HEL cells in the logarithmic growth phase were collected, washed, and resuspended in a final volume of 100 μL in PBS and loaded into a U-100 insulin syringe with an attached 27-gauge needle and injected into the right flank. Approximately 13 days after injection, once tumors were palpable, tumor measurements were collected and mice were randomized into two treatment groups: vehicle control or treatment with velcrin (BAY 2666605), with 10 mice per group. As vehicle for BAY 2666605, we used 5% ethanol in 95% polyethylene glycol-400. Mice in the control group received 5% ethanol in polyethylene glycol-400. Mice were treated with vehicle control or BAY 2666605 (5 mg/kg), which was administered orally, twice daily for 2 weeks, with 2 days off. Tumor size was measured three times per week by caliper and the formula used was (D × d2) × π/6, in which D is the longest diameter and d is the shorter diameter. Body weights and clinical observations were recorded three times per week. For survival, mice were observed daily and compassionately euthanized once the study endpoints were reached (volume limit ≥ 2,000 mm^3^ or weight loss > 20%). The same animal protocol was followed for RNA sequencing (RNA-seq) and Western blot analysis, except there were five animals per treatment group, mice were only treated for 5 days, and tumors were collected 1 hour following the last treatment dose. Tumor material was subsequently processed for SDS-PAGE or RNA-seq analysis. For RNA-seq analysis, tumors from mice bearing HEL flank tumors treated with vehicle control or BAY 2666605 (*n* = 4 per group) were processed into a single-cell suspension for subsequent isolation of RNA with the RNeasy Plus Mini Kit as described by the manufacturer (Qiagen). RNA was reconstituted in RNase-free water.

### Bulk RNA-seq library preparation and sequencing

RNA quality and quantity were assessed by the Bioanalyzer RNA pico chip and Qubit RNA HS assay kit, respectively. One thousand nanogram of RNA was used as input for library construction using Illumina Stranded Total RNA Prep with Ribo-Zero Plus (Illumina, cat. #20040529) according to the manufacturers’ protocol with the following modification: to retain the rRNA, we skipped the rRNA depletion step and started with RNA fragmentation and denaturation directly. The multiplexed libraries were sequenced on NovaSeq X Plus using paired-end 50nt mode. All data were presented as total read counts and fragments per kilobase per million reads. Library preparation, sequencing, and analysis were conducted by the NUSeq Core facility at Northwestern University.

### RNA-seq analysis

The quality of reads, in FASTQ format, was evaluated using FastQC. Reads were trimmed to remove Illumina adapters from the 3′ ends using cutadapt (35). Trimmed reads were aligned to the human genome (hg38) using STAR (36). Read counts for each gene were calculated using htseq-count (37) in conjunction with a gene annotation file for hg38 obtained from Ensembl (http://useast.ensembl.org, RRID: SCR_002344). Normalization and differential expression were calculated using DESeq2 that employs the Wald test (38). The cutoff for determining significantly differentially expressed genes was an FDR-adjusted *P* value less than 0.05 using the Benjamini–Hochberg method. A pathway analysis was performed using Metascape (http://metascape.org, RRID: SCR_016620; ref. 39) to identify significant pathways among the significantly differently expressed genes.

### Statistical analysis

All statistical analyses were performed using GraphPad V.10.0 Software (Prism, RRID: SCR_002798). For *in vitro* studies, three independent experiments were performed, each in triplicate unless otherwise noted, and technical replicates were averaged. Statistical analysis methods for each experiment are described in the figure legends. Statistical significance was found when *P* ≤ 0.05.

## Results

### SLFN12 expression in AML

**Figure 1.**
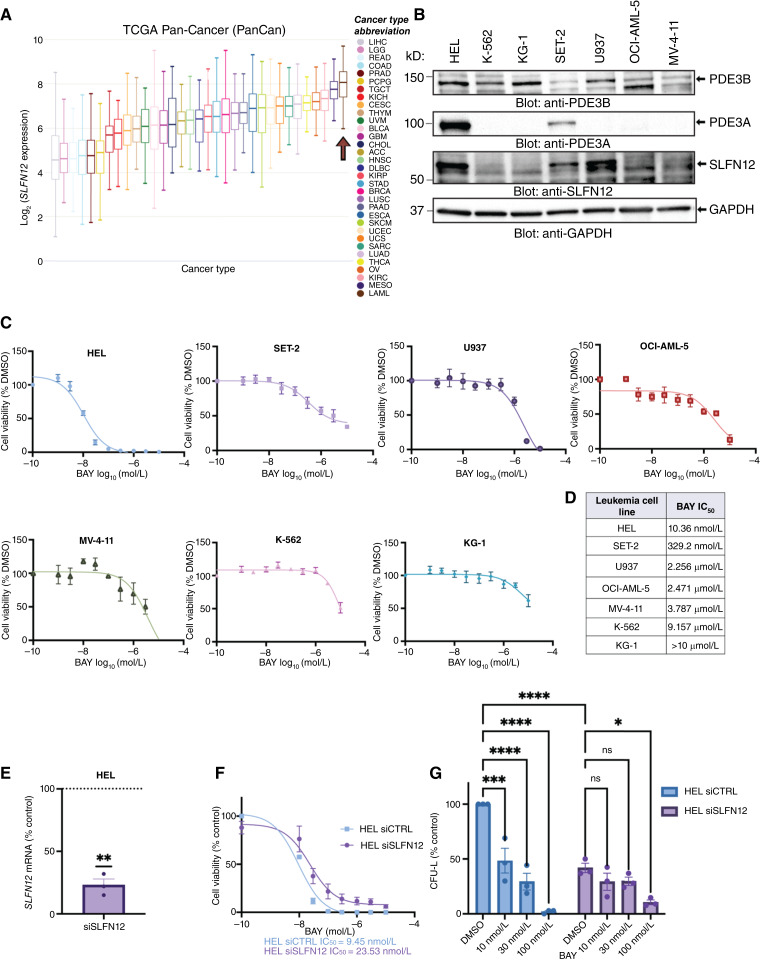
SLFN12 expression and velcrin sensitivity of leukemic lines. **A,** Box plots of *SLFN12* mRNA expression in various cancer types analyzed from the TCGA Pan-Cancer dataset using the University of California Santa Cruz Xena Browser portal. **B,** Immunoblotting analysis of PDE3B, PDE3A, and SLFN12 in established leukemia cell lines (HEL, K-562, KG-1, SET-2, U937, OCI-AML-5, and MV-4-11). **C,** Dose–response curves for cell viability of the indicated leukemia cell lines upon treatment with increasing concentrations of BAY 2666605 for 4 days, assessed by WST-1 assays. For each cell line, data represent means ± SEM of three independent experiments, each done in triplicate. **D,** IC_50_ values for the indicated lines were calculated by fitting a nonlinear regression analysis using the “log (inhibitor) vs. response - three parameters” function in GraphPad Prism software (v.10). **E,***SLFN12* mRNA expression of HEL cells was assessed by qRT-PCR normalized to *GAPDH*, using a non-targeting siRNA as a control. Data are expressed as the percentage of *SLFN12* mRNA expression over control (%) and represent means ± SEM of three independent experiments, each conducted in triplicate. Statistical analysis was performed using a one-sample Student *t* test; **, *P* ≤ 0.01. **F,** Dose–response curve of HEL cells 4 days following transfection with control or *SLFN12*-targeting siRNA and treatment with increasing concentrations of BAY 2666605, as measured by WST-1. Data represent cell viability normalized to siRNA control and displayed as means ± SEM of three independent experiments, each conducted in triplicate. IC_50_ values for the indicated lines were calculated by fitting a nonlinear regression analysis using the “log (inhibitor) vs. response - three parameters” function in GraphPad Prism software (v.10). **G,** Clonogenic assay of HEL cells transfected with control or *SLFN12*-targeting siRNA and treated with the indicated concentrations of BAY 2666605 for 7 days. Leukemic colony formation (CFU-L) was calculated as percent colony formation relative to vehicle control (DMSO-treated cells) and means ± SEM of three independent experiments are presented. Each dot represents an independent experiment. Statistical significance was determined using an ordinary two-way ANOVA, followed by a Tukey multiple comparisons test; ns, not significant, *, *P* ≤ 0.05; ***, *P* ≤ 0.001; ****, *P* ≤ 0.0001. ACC, adrenocortical carcinoma; BAY, BAY 2666605, BLCA, bladder urothelial carcinoma; BRCA, breast invasive carcinoma; CESC, cervical squamous cell carcinoma and endocervical adenocarcinoma; CHOL, cholangiocarcinoma; COAD, colon adenocarcinoma; DLBC, lymphoid neoplasm diffuse large B-cell lymphoma; ESCA, esophageal carcinoma; GBM, glioblastoma multiforme; HNSC, head and neck squamous cell carcinoma; KICH, kidney chromophobe; KIRC, kidney renal clear cell carcinoma; KIRP, kidney renal papillary cell carcinoma; LAML, acute myeloid leukemia; LGG, brain lower grade glioma; LIHC, liver hepatocellular carcinoma; LUAD, lung adenocarcinoma; LUSC, lung squamous cell carcinoma; MESO, mesothelioma; OV, ovarian serous cystadenocarcinoma; PAAD, PANCREATIC adenocarcinoma; PCPG, pheocromocytoma and paraganglioma; PRAD, prostate adenocarcinoma; READ, rectum adenocarcinoma; SARC, sarcoma; SKCM, skin cutaneous melanoma; STAD, stomach adenocarcinoma; TGCT, testicular germ cell tumors; THCA, thyroid carcinoma; THYM, thymoma; UCEC, uterine corpus endometrial carcinoma; UCS, uterine carcinosarcoma; UVM, uveal melanoma.

In initial studies, we used TCGA Pan-Cancer Atlas to assess *SLFN12* mRNA expression across several cancer types. The expression of *SLFN12* was highest in AML compared with all other cancer types included in TCGA Pan-Cancer Atlas (Fig. 1A). We also examined protein expression of SLFN12, PDE3A, and PDE3B in a panel of leukemia cell lines and found that HEL cells exhibit readily detectable amounts of all three proteins (Fig. 1B). Besides HEL, SLFN12 was also readily detectable in U937 and SET-2 cells, and to a lesser extent in the remaining leukemia lines, whereas PDE3A was below detection levels in K-562, KG-1, U937, OCI-AML-5, and MV-4-11 cells. Interestingly, most cell lines expressed either PDE3A or PDE3B, indicating that in all leukemic cell lines tested, at least one of these two phosphodiesterase three isoforms is present in detectable amounts (Fig. 1B).

As SLFN12, PDE3A, and PDE3B were readily detected in HEL cells, we sought to examine the effects of velcrin treatment using BAY 2666605. As shown in Fig. 1C, there was a dose-dependent suppression of HEL cell viability [Fig. 1C (first panel) and D]. Similarly, SET-2 cells, which also exhibit detectable amounts of PDE3A, were sensitive to BAY 2666605 in the nanomolar range [Fig. 1C (second panel) and D]. Leukemia cell lines with undetected PDE3A showed reduced BAY 2666605 sensitivity (Fig. 1B–D). To further validate the role of SLFN12 in velcrin sensitivity, we examined the effects of *SLFN12* knockdown on cell viability and clonogenic capacity following treatment with BAY 2666605. *SLFN12* knockdown in HEL cells (Fig. 1E) resulted in a decrease in their clonogenic capacity, suggesting a potential pro-leukemogenic role for native SLFN12 in AML, although this needs to be confirmed in future studies. Nevertheless, sensitivity to BAY 2666605 was significantly reduced upon *SLFN12* knockdown as assessed by both cell viability and clonogenic assays (Fig. 1F and G). Together, these data indicate that BAY 2666605 sensitivity requires SLFN12 expression in AML. Furthermore, cells that are sensitive to BAY 2666605 depict detectable amounts of PDE3A, whereas cells expressing predominantly PDE3B show reduced sensitivity to velcrin treatment in comparison.

**Figure 2.**
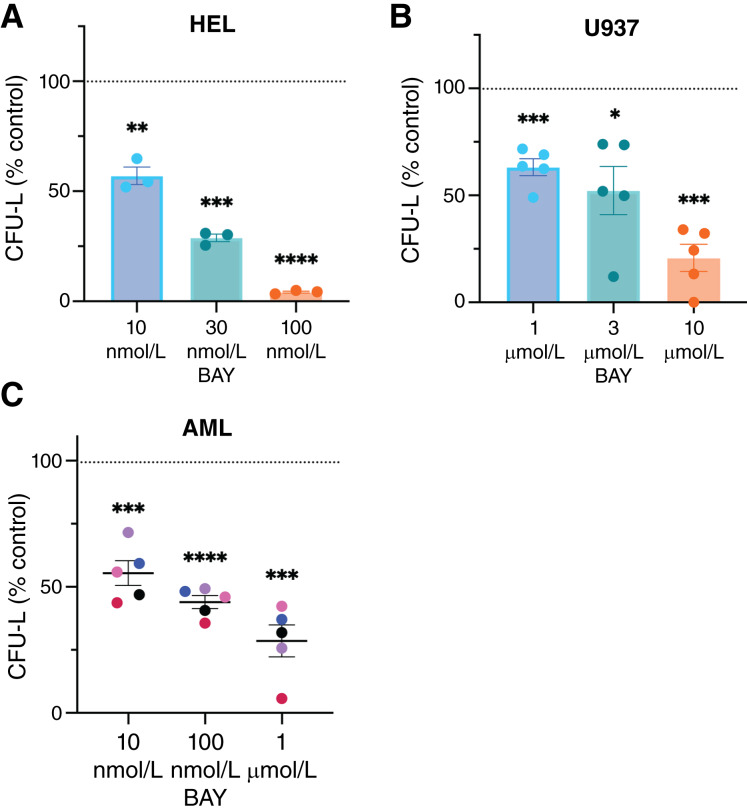
Effects of velcrin treatment on leukemic progenitor colony formation. **A** and **B,** Clonogenic capability of HEL (**A**) or U937 (**B**) cells following treatment with increasing concentrations of BAY 2666605, as noted, for 1 week. Leukemic colony formation (CFU-L) was calculated as percent colony formation relative to vehicle control (DMSO)–treated cells (indicated by the dotted line) and means ± SEM of (**A**) three or (**B**) five independent experiments are presented. Each dot represents an independent experiment. At each concentration, statistical significance was determined using a one-sample Student *t* test compared with 100%; *, *P* ≤ 0.05; **, *P* ≤ 0.01; ***, *P* ≤ 0.001; ****, *P* ≤ 0.0001. **C,** Effects on primary AML-derived leukemic progenitor colony formation. Cells were plated in a methylcellulose culture assay system and treated the same day with BAY 2666605 at the noted concentrations. Leukemic colony formation (CFU-L) was calculated as percent colony formation of BAY 2666605–treated patient samples (2 weeks) relative to vehicle control (DMSO)–treated cells (indicated by the dotted line). Each dot represents an independent experiment. Data are shown as means ± SEM of five independent experiments, as indicated, using primary cells from five different patients with AML. At each concentration, statistical significance was determined using a one-sample Student *t* test compared with 100%; ***, *P* ≤ 0.001; ****, *P* ≤ 0.0001. BAY, BAY 2666605.

Next, we assessed the effects of BAY 2666605 on primitive leukemic progenitors (CFU-L). A statistically significant decrease in both HEL- and U937-derived CFU-L was observed in both lines upon treatment with increasing doses of BAY 2666605 (Fig. 2A and B) in clonogenic assays in methylcellulose. Importantly, treatment with BAY 2666605 also suppressed the growth of primary AML leukemic progenitors (Fig. 2C). However, additional studies to determine the effects of BAY 2666605 on normal human hematopoietic stem/progenitor cells will be required in the future to exclude the possibility that the effects seen on primary AML leukemic progenitors may reflect broad toxicity to hematologic cells.

### Velcrin stabilizes SLFN12 and stimulates PDE3A/B–SLFN12 complex formation, apoptosis, and antileukemic effects *in vitro*

**Figure 3.**
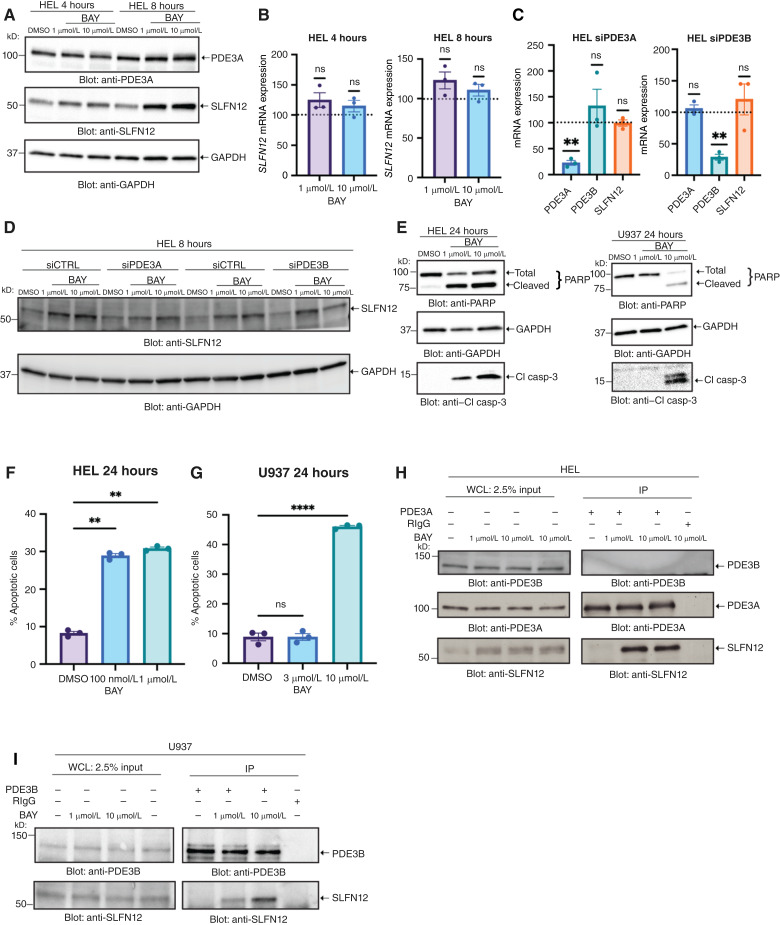
Effects of velcrin treatment on SLFN12 protein levels, PDE3A/B binding, and induction of cell death. **A,** HEL cells were treated with vehicle control (DMSO) or with the indicated doses of BAY 2666605 for 4 or 8 hours. Cell lysates were resolved by SDS-PAGE and immunoblotted with the indicated antibodies. GAPDH is shown as a loading control. **B,***SLFN12* mRNA expression of HEL cells after BAY 2666605 treatment at the indicated concentrations for 4 hours (left) or 8 hours (right). *SLFN12* expression was assessed by qRT-PCR normalized to *GAPDH* and DMSO was used as a vehicle control (indicated by the dotted line). Data represent means ± SEM of three independent experiments, each conducted in triplicate. At each concentration, statistical significance was determined using a one-sample Student *t* test compared with 100%; ns, not significant. **C,** mRNA expression of respective genes in HEL cells transfected with a non-targeting siRNA control (indicated by the dotted line) and siRNA targeting *PDE3A* (left) or *PDE3B* (right). Data are expressed as the percentage of respective mRNA expression over non-targeting siRNA control (%) and represent means ± SEM of three independent experiments, each conducted in triplicate. Statistical significance was determined using a one-sample Student *t* test compared with 100%; ns, not significant, **, *P* ≤ 0.01. **D,** Immunoblot analysis of HEL cells. HEL cells were transfected with respective siRNA for 24 hours (see panel **C**), followed by 8 hours of low (1 μmol/L) or high (10 μmol/L) doses of BAY 2666605, using DMSO as a vehicle control. Cell lysates were resolved by SDS-PAGE and immunoblotted with the indicated antibodies (**E**). HEL (left) or U937 (right) cells were treated with vehicle control (DMSO) or with low (1 μmol/L) or high (10 μmol/L) concentrations of BAY 2666605 for 24 hours. Cell lysates were resolved by SDS-PAGE and immunoblotted with the indicated antibodies. **F** and **G,** Upon treatment with increasing doses of BAY 2666605 for 24 hours, HEL (**F**) and U937 (**G**) cells were stained with annexin V/propidium iodide and subjected to flow cytometry. The percentage of apoptotic cells for HEL (**F**) and U937 (**G**) cells treated with increasing BAY 2666605 concentrations is shown as mean ± SEM of three independent experiments. Statistical significance was determined using a one-way ANOVA, followed by a Dunnett multiple comparisons test to adjust for multiple comparisons for each dose vs. control; **, *P* ≤ 0.01; ****, *P* ≤ 0.0001. **H** and **I,** Co-immunoprecipitation analysis of SLFN12 interaction with (**H**) PDE3A in HEL cells or (**I**) PDE3B in U937 cells. HEL and U937 cells were treated with low (1 μmol/L) or high (10 μmol/L) concentrations of BAY 2666605 for 24 hours. DMSO was used as vehicle control. Cell lysates were used for immunoprecipitation with anti-PDE3A and anti-PDE3B antibodies or control rabbit immunoglobulin, as indicated. **H,** After probing for PDE3A, the membrane was stripped and reprobed with antibody for PDE3B. BAY, BAY 2666605; Cl casp-3, cleaved caspase-3; IP, immunoprecipitation; RIgG, rabbit immunoglobulin; WCL, whole-cell lysate.

To precisely define the mechanisms accounting for the antileukemic properties of BAY 2666605, we examined its effects on SLFN12 protein stability. We found stabilization of SLFN12 protein expression as early as 4 and 8 hours after treatment with BAY 2666605 (Fig. 3A), whereas PDE3A protein levels remained unchanged, suggesting a SLFN12-specific stabilization effect at the protein level (Fig. 3A), whereas mRNA levels remained largely unaffected (Fig. 3B). To further dissect the role of PDE3 isoforms in SLFN12 stabilization, we transfected HEL cells with siRNAs targeting *PDE3A* [Fig. 3C (left)] or *PDE3B* [Fig. 3C (right)] and assessed relative mRNA expression of *PDE3A*, *PDE3B*, and *SLFN12*. No obvious effects on *SLFN12* mRNA levels were observed after knockdown of either *PDE3* isoform, suggesting that protein stabilization is a direct effect of BAY 2666605 treatment. Remarkably, knockdown of PDE3A resulted in impaired SLFN12 protein stabilization (Fig. 3D), corroborating the role of PDE3A in SLFN12 protein stabilization following velcrin treatment. Further studies revealed an increase of apoptotic markers such as cleaved PARP and cleaved caspase-3 in HEL [Fig. 3E (left)] and U937 [Fig. 3E (right)] cells, consistent with induction of apoptosis following SLFN12 stabilization using BAY 2666605. Similarly, we found that compared with DMSO-treated control cells, there was an increase in the percentage of early- and late-stage apoptotic cells for both HEL (Fig. 3F; Supplementary Fig. S1) and U937 (Fig. 3G; Supplementary Fig. S1) cells following BAY 2666605 treatment.

Previous studies have shown that PDE3A can form a heterotetrameric complex with SLFN12 dimers (26, 40, 41). To investigate whether endogenous SLFN12 interaction with PDE3A/B is associated with the induction of antileukemic effects, HEL or U937 cells were treated with BAY 2666605, followed by co-immunoprecipitation experiments 24 hours after treatment. SLFN12 and PDE3A co-immunoprecipitated upon velcrin treatment (Fig. 3H), suggesting that in cell lines expressing high SLFN12 and PDE3A protein, the formation of the SLFN12–PDE3A complex is induced by velcrin treatment as shown in previous models (26, 40, 41). However, most PDE3A-deficient leukemia cell lines that we tested still responded to treatment with BAY 2666605, suggesting a potential PDE3A-compensatory pathway for velcrin sensitivity. We hypothesized that in the absence of PDE3A, another PDE3 isoform, PDE3B, may compensate for velcrin-induced heterotetrameric complex formation and subsequent antineoplastic effects in AML (27). To examine this hypothesis, we performed co-immunoprecipitation experiments in U937 cells, a leukemia cell line with undetected PDE3A protein expression but high PDE3B expression. We found that in these cells, SLFN12 co-immunoprecipitated with PDE3B 24 hours following velcrin treatment (Fig. 3I), suggesting that in the absence of PDE3A, SLFN12 associates with PDE3B in a velcrin-dependent manner. Notably, there were no detectable amounts of PDE3B in PDE3A immunoprecipitates (Fig. 3H), suggesting that the PDE3 isoforms associate with SLFN12 in a mutually exclusive manner. Altogether, these findings suggest that BAY 2666605 treatment promotes SLFN12–PDE3A and/or SLFN12–PDE3B complex formation, resulting in SLFN12 stabilization and induction of apoptosis.

**Figure 4.**
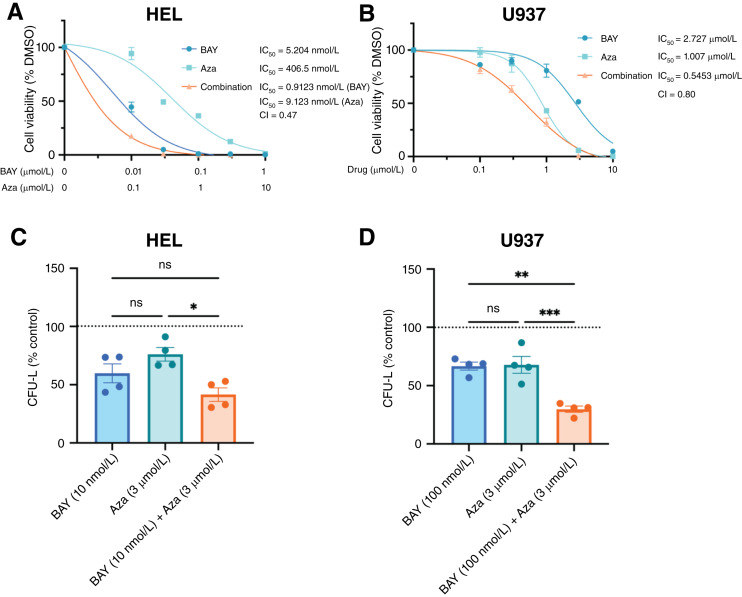
Antileukemic effects of the velcrin–Aza combination. **A** and **B,** Cell viability of HEL (**A**) or U937 (**B**) cells treated with DMSO (vehicle control) or with increasing concentrations of BAY 2666605 (BAY), Aza, or the combination of BAY 2666605 with Aza as measured by WST-1 cell viability assay. Data are presented as the means ± SEM of three independent experiments, each conducted in triplicate, and are expressed as percent cell viability relative to vehicle control (DMSO)–treated cells. IC_50_ values for the indicated cell lines were calculated by fitting a nonlinear regression analysis using the “log (inhibitor) vs. response - three parameters” function in GraphPad Prism software (v.10). CI was calculated using Compusyn software, in which CI < 1 is synergistic, CI > 1 is antagonistic, and CI = 1 is additive. **C** and **D,** Clonogenic capability of (**C**) HEL or (**D**) U937 cells plated in a methylcellulose culture assay system in the presence of BAY 2666605, Aza, or the combination of BAY 2666605 with Aza, relative to vehicle control (DMSO; indicated by the dotted line) at the indicated concentrations. The percent of leukemic colony formation (CFU-L) is shown relative to vehicle control (DMSO)–treated cells and represents mean ± SEM of four independent experiments. Each dot represents an independent experiment. Statistical significance was determined using a one-way ANOVA, followed by a Tukey multiple comparisons test to correct for pairwise comparisons; ns, not significant; *, *P* ≤ 0.05; **, *P* ≤ 0.01; ***, *P* ≤ 0.001. BAY, BAY 2666605.

The hypomethylating agent Aza is used alone or in combination with the BCL2 inhibitor venetoclax in the treatment of patients with AML (42), but its efficacy is limited and patients eventually relapse after treatment. Considering the high expression levels of SLFN12 in AML cells, we sought to determine the effects of the combination of BAY 2666605 with Aza treatment in AML lines. Initially, we conducted WST-1 cell viability assays and observed synergistic suppressive effects in both HEL (Fig. 4A; CI = 0.47) and U937 (Fig. 4B; CI = 0.80) cells. Similarly, results from colony formation assays (Fig. 4C and D) indicate a decrease in clonogenic capacity in leukemic precursors following treatment with both Aza and BAY 2666605 as compared with either of the treatments alone.

### BAY 2666605 prolongs survival in an AML xenograft model

**Figure 5.**
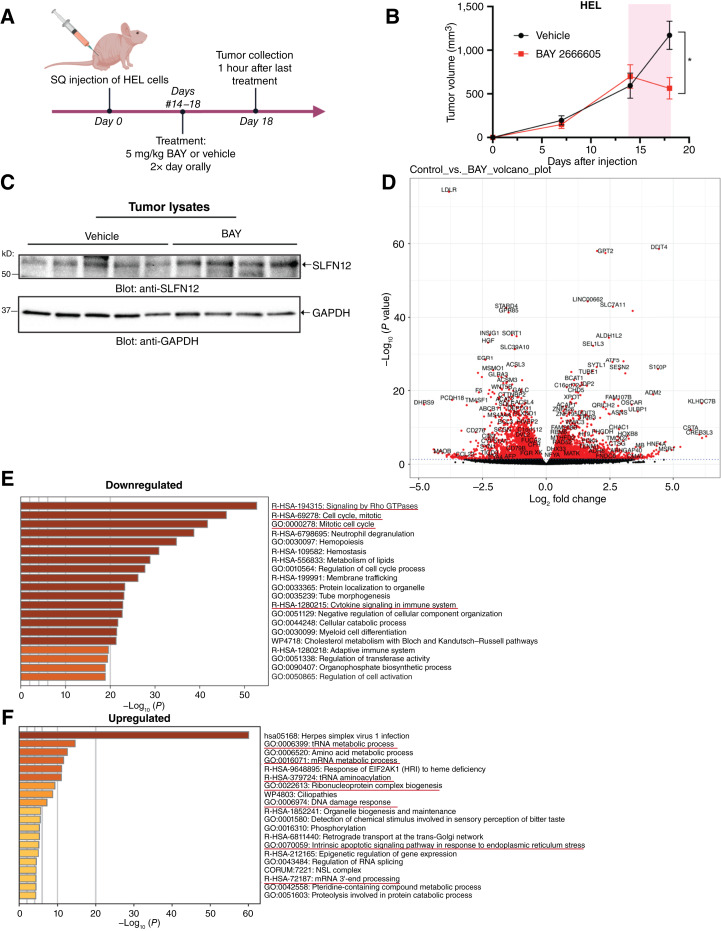
Effects of velcrin treatment on SLFN12 expression *in vivo*. **A,** Schematic representation of the treatment regimen used for the xenograft AML mouse model using BAY 2666605 or vehicle control. **B,** Tumor growth curves of nude (NU/NU) mice treated and challenged with HEL cells. Each point represents the mean tumor volume ± SEM per group for vehicle control (5% ethanol with 95% polyethylene glycol-400)– and BAY 2666605–treated mice (*n* = 5 for control; *n* = 4 for treated). Pink bar indicates days in which mice were treated with BAY 2666605 or vehicle control. Tumor growth from the start of treatment (day 14) until the end of treatment (day 18) was analyzed using a mixed-effects linear regression model. Tumor volume was log-transformed to satisfy the normality assumption. Treatment group, time, and their interaction were included as fixed effects and mice as a random effect. The interaction was statistically significant (*P* < 0.001). The *P* value comparing treatments at day 18 is reported (*, *P* = 0.011). **C,** Tumor lysates from the experiment shown in panel (**B**) were subjected to SDS-PAGE and probed for SLFN12 protein expression. **D,** Tumor samples from the same experiment (*n* = 4 per group) were subjected to RNA-seq. Volcano plot shows the differentially up- or downregulated genes between the velcrin treatment group and the vehicle control group. Genes deemed statistically significant are highlighted in red. Data are from four biological replicates per group (see also Supplementary Fig. S2). **E** and **F,** Gene ontology (GO) analysis of differentially expressed genes that were (**E**) downregulated or (**F**) upregulated in mouse tumors treated with BAY 2666605 compared with vehicle control–treated mice. Relevant pathways associated with differentially expressed genes are underlined in red. BAY, BAY 2666605; SQ, subcutaneous. [**A,** Created in BioRender. Guillen Magana, J. (2025) https://BioRender.com/aqz79jx.]

To investigate the effects of BAY 2666605 treatment *in vivo*, we employed a xenograft AML mouse model, treating mice with either vehicle (*n* = 5) or BAY 2666605 (*n* = 4; Fig. 5A). Notably, just 5 days of BAY 2666605 treatment led to a dramatic reduction in tumor growth compared with vehicle-treated control mice (Fig. 5B). To define the *in vivo* mechanism of such effects, we examined whether SLFN12 stabilization occurred *in vivo*. We detected elevated SLFN12 protein amounts in tumors from velcrin-treated mice, indicative of SLFN12 protein stabilization *in vivo* after a single treatment cycle with BAY 2666605 (Fig. 5C). We also performed RNA-seq analysis in order to identify differentially expressed genes following velcrin treatment *in vivo* (Fig. 5D; Supplementary Fig. S2). BAY 2666605 treatment significantly increased the expression of 2,073 genes and significantly decreased the expression of 2,779 genes (Fig. 5D). Gene ontology analyses (Fig. 5E and F) revealed that genes upregulated by BAY 2666605 treatment are associated with tRNA metabolic processes, DNA damage response, and intrinsic apoptotic signaling pathways (Fig. 5F). These findings are consistent with previous *in vitro* work demonstrating that SLFN12’s biological function as a tRNase is enhanced upon velcrin treatment (43). Additionally, changes in mRNA processes, such as mRNA metabolism and mRNA 3′ end processing (Fig. 5F), suggest potential regulation at the mRNA level following velcrin treatment and should be further investigated in future studies.

**Figure 6.**
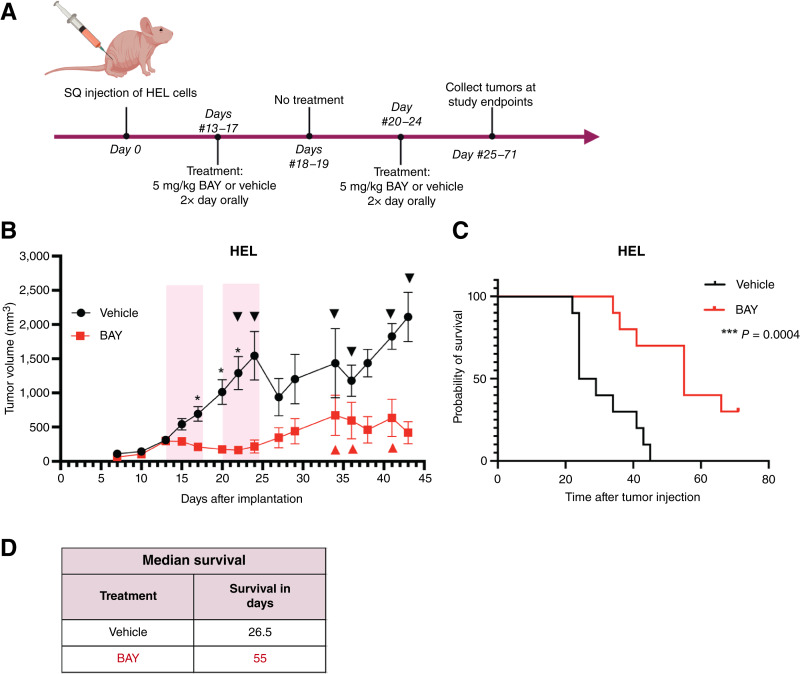
Antileukemic activity of BAY 2666605 *in vivo*. **A,** Schematic representation of the experimental approach for the treatment regimen used for the xenograft AML mouse model. **B,** Tumor volumes (means ± SEM) are shown for mice treated with either vehicle control (5% ethanol with 95% polyethylene glycol-400) or BAY 2666605 (5 mg/kg; *n* = 10 per group). Pink bars indicate treatment window. Tumor growth from the start of treatment (day 13) until the end of treatment (day 24) was analyzed using a mixed-effects linear regression model. Tumor volume was log-transformed to satisfy the normality assumption. Treatment group, time, and their interaction were included as fixed effects and mice as a random effect. The interaction was statistically significant (*P* < 0.0001). The *P* values comparing treatments at each time point were adjusted for multiple comparisons using the Holm–Šídák method; *, *P* ≤ 0.05. Arrowheads indicate when mice were euthanized because of meeting study endpoints after tumor volume measurement. **C,** Kaplan–Meier survival analysis for mice treated with BAY 2666605 vs. vehicle control. Mice were compassionately euthanized once tumor volume was ≥2,000 mm^3^ or weight loss was greater than 20%; ***, *P* = 0.0004 using a Mantel–Cox (log-rank) test. **D,** Table summarizing estimated median survival (in days) of mice from each treatment group. BAY, BAY 2666605; SQ, subcutaneous. [**A,** Created in BioRender. Guillen Magana, J. (2025) https://BioRender.com/yw4tt0f.]

We next used the xenograft AML mouse model to determine whether the effects of BAY 2666605 on SLFN12 stabilization and changes in gene expression *in vivo* correlate with differences in leukemic cell growth and survival (Fig. 6A). Mice were treated with two cycles of BAY 2666605 or vehicle and were monitored for body weight, tumor growth, and survival until study endpoints (Fig. 6A). We observed a significant reduction in tumor volume after only one treatment cycle with BAY 2666605 in comparison with vehicle-treated mice. This difference became more pronounced over time and persisted after treatment (Fig. 6B). There were no obvious effects on body weight, indicating low toxicity and favorable tolerability at the given dose (Supplementary Fig. S3). However, it is important to note that there is no known mouse homolog for *SLFN12*; therefore, interpretations of toxicity in this study are limited. Nonetheless, mice that received BAY 2666605 treatment had a major survival benefit, with a median survival of 55 days, as compared with the control group for which median survival was estimated at only 26.5 days (Fig. 6C and D).

## Discussion

Our study provides evidence for a novel role of SLFN12 in AML and defines it as a potential leukemic therapeutic target that can be modulated using velcrin treatment. Previous studies have determined that SLFN12 undergoes constitutive degradation via the ubiquitin–proteasome system (25, 44). Thus, in order to efficiently promote antineoplastic effects, the SLFN12 protein must first be stabilized. Previous work has demonstrated that treatment with velcrins can stabilize SLFN12 in solid tumors, albeit with different potencies (40, 44, 45). Our study reveals stabilization of SLFN12 protein as early as 4 hours after treatment at very low concentrations of BAY 2666605 in leukemia cells both *in vitro* and *in vivo*, resulting in potent antileukemic responses and forming the basis for future clinical–translational efforts in that direction.

Velcrins are a class of small molecules that were initially discovered as a subset of PDE3 inhibitors (46). Subsequent investigations demonstrated that velcrins act as molecular glues with high potency in promoting complex formation between PDE3A and SLFN12 (40, 47, 48). In solid tumors, the protein–protein interaction between PDE3A and SLFN12 is well established in several cancer types, and the crystallized structure for this interaction has been resolved (41, 44, 49). Another PDE3 isoform, PDE3B, shares a conserved catalytic domain with PDE3A (50). Given that PDE3A functions primarily as a scaffold rather than a substrate-selective enzyme, it is possible that PDE3B may serve as a compensating protein for the stabilization of SLFN12 (41). However, most studies exploring a potential role for PDE3B in velcrin sensitivity focused on a highly unstable velcrin, DNMDP (47, 48). During the development of BAY 2666605, a derivative of DNMDP, no major difference in selectivity for PDE3 isoforms was observed when tested against PDE3A and PDE3B (31). Here, we provide evidence that physiologic levels of PDE3B, even in the absence of PDE3A, can be sufficient for heterotetrameric complex formation with SLFN12 to induce velcrin-mediated antineoplastic effects, further demonstrating a potential role for PDE3B in velcrin sensitivity in leukemia.

It should be noted that a potential velcrin-independent pro-leukemogenic role for SLFN12 was observed following siRNA knockdown of SLFN12 as assessed by clonogenic assays (Fig. 1E–G). However, our experiments suggest that velcrin treatment of SLFN12-expressing AML cells results in complex formation between SLFN12 and PDE3 (Fig. 3H and I), leading to SLFN12 stabilization (Fig. 3A–D) and induction anti-leukemogenic effects (Fig. 3E and F). Thus, although SLFN12 in its unbound form may support some leukemic function, velcrin-induced complex formation is required for antileukemic effects. This hypothesis is further supported by our observations that knocking down *SLFN12* reduces sensitivity to BAY 2666605 in both cell viability and clonogenic assays. Although these observed effects are velcrin dependent, we cannot entirely exclude a velcrin-independent role for SLFN12 in leukemogenesis as the biological role of SLFN12 in its native form remains largely unexplored.

Of the known biological roles of SLFN12, significant attention has previously been directed toward understanding SLFN12 as a tRNase and rRNase, augmenting its biological function through velcrin treatment (43). Interestingly, our RNA-seq findings from an *in vivo* model suggest several genes/pathways involved at the transcriptional level that should be further explored. Of note, among the top upregulated genes was DNA damage–inducible transcript-4, a negative regulator of the mTOR pathway, and a protein that has been implicated in contributing to AML (51). This suggests a possible new mechanism for velcrin-mediated apoptosis and antineoplastic effects which warrants validation in future studies and, particularly, the contribution of SLFN12 in this process. In regard to downregulated genes by velcrin treatment *in vivo*, our gene ontology analysis revealed that there were genes associated with pathways involving Rho GTPases, which have been previously implicated in the initiation, progression, growth, and survival of hematologic malignancies (52). Additionally, among the top biological effects associated with downregulated genes were changes in the cell cycle (mitosis) and cytokine signaling. Aberrant cytokine signaling in AML is thought to majorly contribute to AML tumorigenesis and progression, with AML-promoting cytokines such as IL1β, IL6, and CXCL12 being investigated for the development of targeted therapies (53). These observations support future studies to better define the molecular mechanisms underlying SLFN12’s role in AML after velcrin treatment.

Based on previous work in solid tumors such as glioblastoma, only PDE3A and SLFN12 are considered as biomarkers for treatment with velcrins (26). We provide evidence that even in less sensitive leukemia cell lines, BAY 2666605 remains effective at relatively low doses and enhances Aza antileukemic effects, suggesting that this combinatorial strategy could be particularly beneficial for patients with lower PDE3A and higher PDE3B expression. It is also important to note that currently, targeted therapies for FLT3, isocitrate dehydrogenase-1/2, and BCL2, which have driven the development of FLT3 inhibitors, isocitrate dehydrogenase inhibitors, and venetoclax, respectively (54), have limited applicability. Approximately half of patients with AML lack targetable abnormalities or develop resistance mechanisms (55). Unlike most targeted therapies aimed at enzyme inhibition or protein degradation, velcrins stabilize SLFN12, offering a novel therapeutic strategy based on a gain-of-function mechanism. Hence, this potential therapeutic approach is distinct from most current AML treatments that nonspecifically target general cellular functions.

## Supplementary Material

Supplementary Figure S1 Figure S1. Dot plot of HEL and U937 cells undergoing apoptosis.

Supplementary Figure S2 Figure S2. PCA of tumor samples and Heatmap of DEGs.

Supplementary Figure S3 Figure S3. Stable body weight and tumor volume post-BAY treatment in vivo.

Supplementary Table S1 Table S1. Key Resources Table

## Data Availability

Original files for RNA-seq data have been uploaded to the NIH Gene Expression Omnibus web portal under the accession number GSE296195. For original data, please contact L. C. Platanias (l-platanias@northwestern.edu) or F. Eckerdt (frank.eckerdt@northwestern.edu).
