# Supplementary material for: Schlafen 12 Modulation and Targeting in Acute Myeloid Leukemia
Source: Cancer Res Commun. 2025 Nov 17;5(11):2012–24. doi: 10.1158/2767-9764.CRC-25-0283 (PMC12620962; doi:10.1158/2767-9764.CRC-25-0283)
Supplement: Supplementary Figure S1 — Figure S1. Dot plot of HEL and U937 cells undergoing apoptosis. [file crc-25-0283_supplementary_figure_s1_suppsf1.docx]

**Supplementary Figure S1**


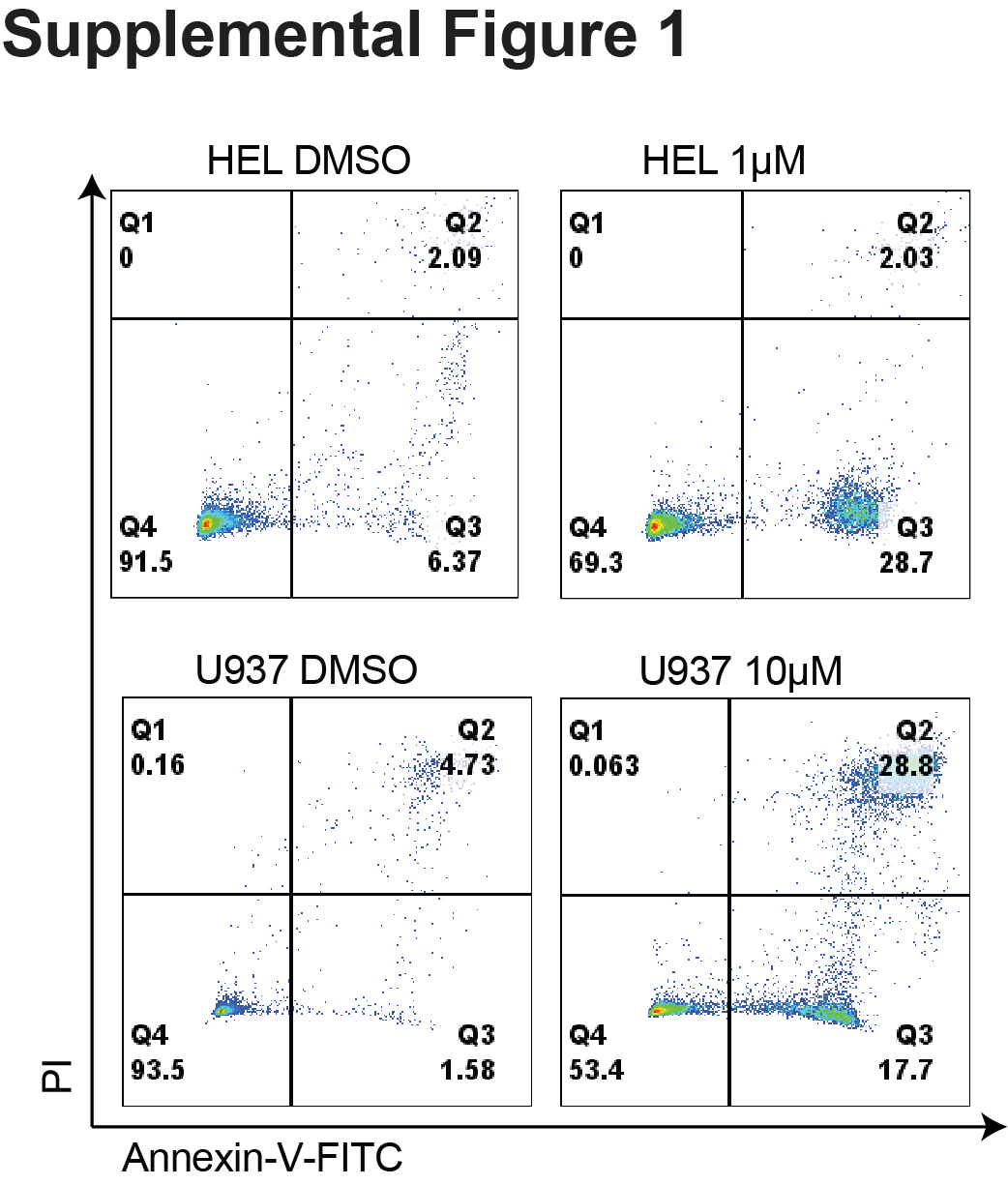


**Supplementary Figure S1: Dot plot of HEL and U937 cells undergoing apoptosis.**

Representative dot plots from experiments in Fig. 3F-G of HEL (top panels) or U937 (bottom panels) treated with DMSO (left panels) or BAY 2666605 (right panels) were generated using FlowJo. Dots represent cells stained with apoptosis markers Annexin-V-FITC, Propidium Iodide (PI), or both. Quadrant 4 (Q4) represents live cells (Annexin V and PI negative), Q3 represents cells in early apoptosis (Annexin V positive and PI negative), Q2 represents cells in late apoptosis (Annexin V and PI positive) and necrotic cells are shown in Q1 (Annexin V negative and PI positive).
