# Supplementary material for: Schlafen 12 Modulation and Targeting in Acute Myeloid Leukemia
Source: Cancer Res Commun. 2025 Nov 17;5(11):2012–24. doi: 10.1158/2767-9764.CRC-25-0283 (PMC12620962; doi:10.1158/2767-9764.CRC-25-0283)
Supplement: Supplementary Figure S2 — Figure S2. PCA of tumor samples and Heatmap of DEGs. [file crc-25-0283_supplementary_figure_s2_suppsf2.docx]

**Supplementary Figure S2**


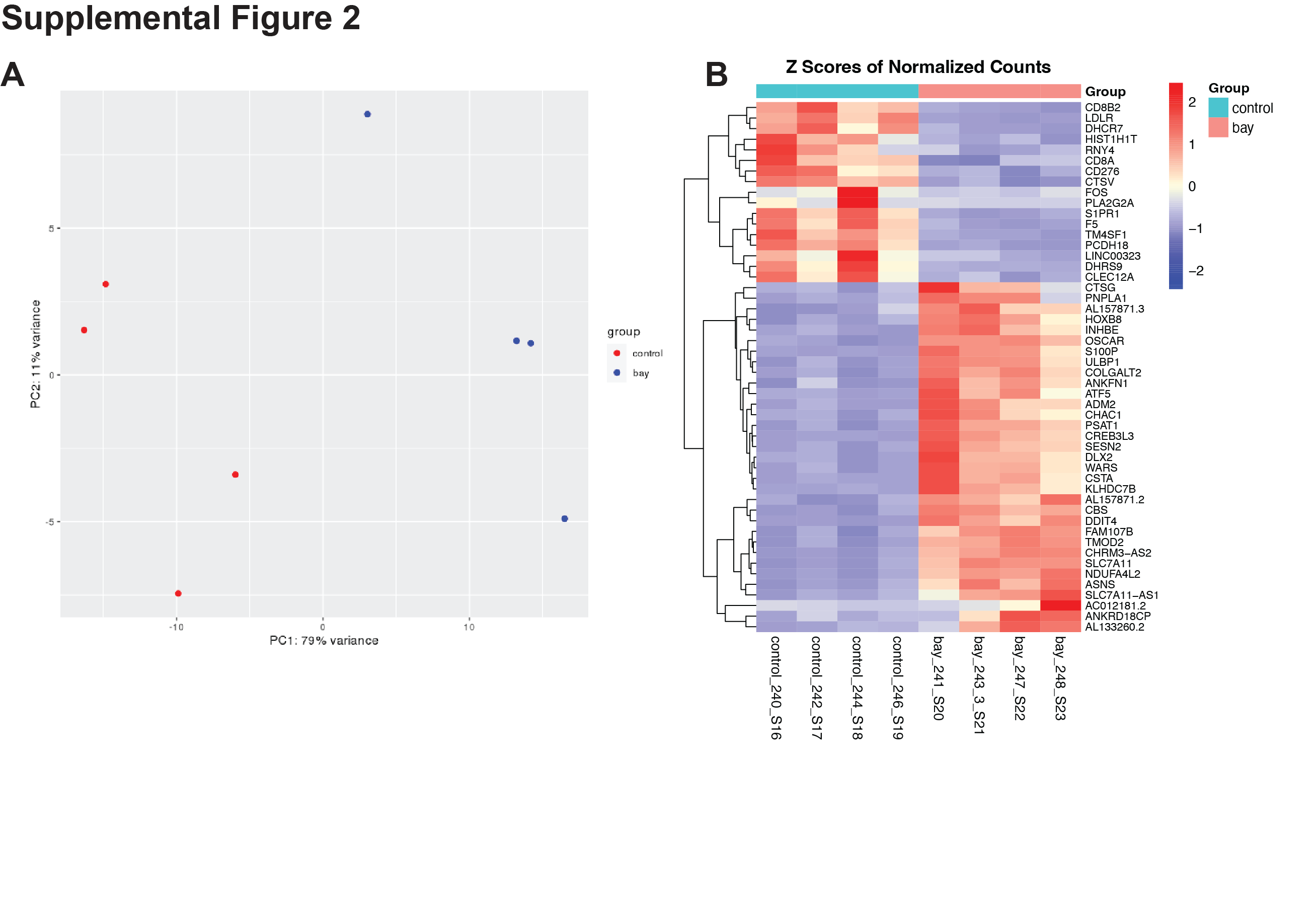


**Supplementary Figure S2: PCA of tumor samples and Heatmap of DEGs.**

Extended analysis of RNA-sequencing from experiment in Fig. 5. **A.** Principal component analysis (PCA) of individual tumor RNA samples analyzed by RNA-sequencing analyses. The PCA plot shows separation between the control group and the BAY treated group and displays reproducibility within each group. Four biological replicates per experimental group cluster together for control mice (labeled in red) and mice treated with BAY 2666605 (labeled in blue). **B.** Heatmap analysis of significantly upregulated and downregulated genes in control (teal, “control”) and BAY 2666605-treated mice bearing HEL-derived tumors (pink, “bay”). Data are from 4 biological replicates per group.
