# Supplementary material for: Schlafen 12 Modulation and Targeting in Acute Myeloid Leukemia
Source: Cancer Res Commun. 2025 Nov 17;5(11):2012–24. doi: 10.1158/2767-9764.CRC-25-0283 (PMC12620962; doi:10.1158/2767-9764.CRC-25-0283)
Supplement: Supplementary Figure S3 — Figure S3. Stable body weight and tumor volume post-BAY treatment in vivo. [file crc-25-0283_supplementary_figure_s3_suppsf3.docx]

**Supplementary Figure S3**
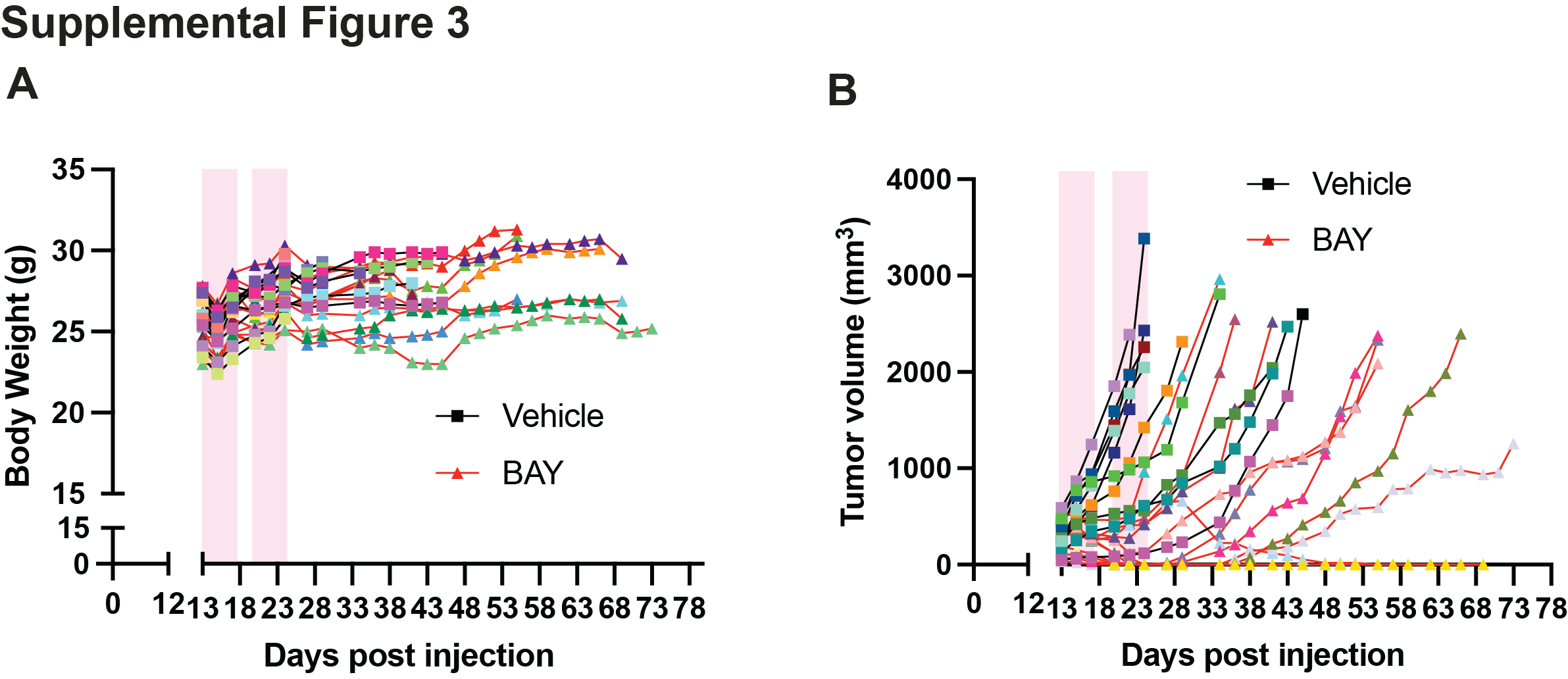


**Supplementary Figure S3: Stable body weight and tumor volume post-BAY treatment *in vivo*.**

Spaghetti plot of mice from experiments shown in Fig. 6. Body weights (A) and individual tumor volumes (B) for mice treated with vehicle-control (represented by squares, connected by black lines) or BAY 2666605 (represented by triangles, connected by red lines) over time are shown. Each color represents individual mice. Pink bars indicate treatment windows.
