# Supplementary material for: Schlafen 12 Modulation and Targeting in Acute Myeloid Leukemia
Source: Cancer Res Commun. 2025 Nov 17;5(11):2012–24. doi: 10.1158/2767-9764.CRC-25-0283 (PMC12620962; doi:10.1158/2767-9764.CRC-25-0283)
Supplement: Supplementary Table S1 — Table S1. Key Resources Table [file crc-25-0283_supplementary_table_s1_suppst1.docx]

| **Reagent** | **Company** | **Identifier** |
| --- | --- | --- |
| **Chemicals/Reagents** | | |
| 10x Tris/Glycine/SDS running buffer | National Diagnostics | Cat#: EC-870 |
| 5-azacitidine (AZA) | TargetMol | Cat#: T1272 |
| BAY 2666605 | MedChem Express | Cat#: HY-145924 Purity |
| Biorad Clarity ECL Western Blotting Substrate | BioRad | Cat#: 1705061 |
| Biorad Clarity Max ECL Western Blotting Substrate | BioRad | Cat#: 1705062 |
| BioRad Criterion TGX Precast 4-15% Gels | BioRad | 12-well Cat#: 5671083 |
| DMEM | Gibco | Cat#: 11965118 |
| FBS | Sigma | Cat#: F2442-500ML |
| GE Healthcare Amersham™ ECL™ | GE Healthcare | Cat#: 45-000-875 |
| Gentamicin | Sigma | Cat#: G1397-10ml |
| GM-CSF | PeproTech/ Thermo Fisher Scientific | Cat#: 300-03-50UG |
| HyBlot CL Autoradiography Film | Thomas Scientific | Cat#: 1141J52 |
| Lane Marker Reducing Sample Buffer 5x | Thermo Fisher Scientific | Cat#: 39000 |
| IMDM | Thermo Fisher Scientific | Cat#: 12440061 |
| MEM alpha | Gibco | Cat#: 12571063 |
| MethoCult™ H4434 Classic | Stem Cell Technologies | Cat#: 04434 |
| Non-fat dry milk | BioRad | Cat#: 1706404 |
| phosphatase inhibitor cocktail set I | EMD Millipore | Cat#: 524624 |
| Precision Plus Protein Dual Color Standards | BioRad | Cat#: 1610374 |
| Precision Plus Protein WesternC Standards BioRad | BioRad | Cat#: 1610385 |
| protease inhibitor cocktail set III | EMD Millipore | Cat#: 539134 |
| RPMI | Gibco | Cat#: 11875119 |
| Sodium bicarbonate 7.5% | Gibco | Cat#: 25080094 |
| sodium orthovanadate | New England Biolabs | Cat#: P0758S |
| Sodium pyruvate 100 mM | Gibco | Cat#: 11965118 |
| WesternSure® PREMIUM Chemiluminescent Substrate | LiCORbio | Cat#: 926-95000 |
| WST-1 | Hoffman LaRoche / Sigma | Cat#: 5015944001 |
| **Experimental Models: Cell Lines** | | |
| HEL | ATCC | Cat#: TIB-180  RRID: CVCL_0001 |
| K-562 | ATCC | Cat#: CCL-243  RRID: CVCL_0004 |
| KG-1 | ATCC | Cat#: CCL-246  RRID: CVCL_0374 |
| MV-4-11 | ATCC | Cat#: CRL-9591  RRID: CVCL_0064 |
| OCI-AML-5 | ATCC | Cat#: ACC-247  RRID: CVCL_1620 |
| SET-2 | DSMZ | Cat#: ACC-608  RRID:CVCL_2187 |
| U937 | ATCC | Cat#: CRL-1593.2  RRID: CVCL_0007 |
| **Experimental Models: Mice** | | |
| NU/NU mice | Charles River laboratories | Strain: 088  RRID: IMSR_CRL:088 |
| **Taqman Probes** | | |
| *GAPDH* | Thermo Fisher Scientific | Cat#: 4331182  AssayID: Hs02758991_g1 |
| *PDE3A* | Thermo Fisher Scientific | Cat#: 4331182  AssayID: Hs01012698_m1 |
| *PDE3B* | Thermo Fisher Scientific | Cat#: 4331182  AssayID: Hs00265322_m1 |
| *SLFN12* | Thermo Fisher Scientific | Cat#: 4351372  AssayID: Hs01049939_m1 |
| **ON-TARGETplus siRNA** | | |
| Non-targeting Control Pool | Horizon | Cat#: D-001810-10-20 |
| *SLFN12* | Horizon | Cat#: L-018142-02-0020 |
| *PDE3A* | Horizon | Cat#: L-007645-00-0005 |
| *PDE3B* | Horizon | Cat#: L-007646-00-0005 |
| **Antibodies** | | |
| Anti-Mouse IgG (H + L)-HRP Conjugate | BioRad | Cat#: 1706516  RRID: AB_11125547 |
| Anti-Rabbit IgG (Light-Chain Specific) (D4W3E) mAb (HRP Conjugate) | Cell Signaling | Cat#: 93702S  RRID:AB_2800208 |
| Anti-rabbit IgG HRP-linked antibody | Cell Signaling | Cat#: 7074S  RRID: AB_2099233 |
| Anti-rabbit IgG polyclonal antibody isotype-control | Protein Tech | Cat#: 30000-0-AP  RRID: AB_2819035 |
| cl-CASP-3 | Cell Signaling | Cat#: 9661S  RRID: AB_2341188 |
| GAPDH | MilliporeSigma | Cat#: MAB374  RRID:AB_2107445 |
| PARP | Cell Signaling | Cat#: 9542S  RRID: AB_2160739 |
| PDE3A | Bethyl | Cat#: A302-740A  RRID: AB_10634214 |
| PDE3B | Bethyl | Cat#: A302-743A  RRID: AB_10631431 |
| SLFN12 | ABCAM | Cat#: ab234418  RRID: N/A |
| **Software and Algorithms** | | |
| Adobe Illustrator | Adobe | <http://www.adobe.com/products/illustrator.html>  RRID:SCR_010279 |
| Adobe Photoshop | Adobe | <https://www.adobe.com/products/photoshop.html>  RRID:SCR_014199 |
| Biorad Image Lab | Biorad | <https://www.bio-rad.com/en-us/product/image-lab-software?ID=KRE6P5E8Z> |
| CompuSyn Software | ComboSyn | <https://www.combosyn.com/index.html> |
| Ensembl | Ensemble | <http://www.ensembl.org/>  RRID: SCR_002344 |
| Excel | Microsoft | N/A |
| FlowJo | BD Biosciences | <https://www.flowjo.com/solutions/flowjo>  RRID:SCR_008520 |
| Gen5 | Agilent Biotek | <https://www.agilent.com/en/product/microplate-instrumentation/biotek-instruments>  RRID:SCR_017317 |
| GraphPad Prism | GraphPad software Inc | <https://www.graphpad.com/scientific-software/prism/>  RRID:SCR_002798 |
| LI-COR Empiria Studio | LI-CORbio | <https://www.licorbio.com/empiria-studio>  RRID:SCR_022512 |
| Metascape | Metascape | <http://metascape.org>  RRID: SCR_016620 |
| University of California Santa Cruz (UCSC) Xena browser | UCSC | <https://xena.ucsc.edu/>  RRID: SCR_018938 |
| **Commercial Assays** | | |
| BD Pharmingen™ FITC Annexin V Apoptosis Detection Kit I | BD Biosciences | Cat#: 556547 |
| Bicinchonic acid assay | Pierce | Cat#: 23223  Cat#: 23224 |
| Bio-Rad Protein Assay Kit II | BioRad | Cat#: 5000002 |
| CD117 MicroBead Kit | Miltenyi Biotec | Cat#: 130-091-332 |
| EasySep magnetic column | Stem Cell Technologies | Cat#: 18000 |
| EasySep™ Human CD34 Positive Selection Kit II | Stem Cell Technologies | Cat#: 17856 |
| High-Capacity cDNA Reverse Transcription Kit | Thermo Fisher Scientific | Cat#: 4368814 |
| Lonza Cell Line Nucleofector® Kit V | Lonza | Cat#: VCA-1003 |
| MycoAlert Mycoplasma detection kit | Lonza | Cat#: LT07-218 |
| RNeasy Mini Kit | Qiagen | Cat#: 74106 |
| Trans-Blot Turbo RTA Midi 0.45 µm LF PVDF Transfer Kit | BioRad | Cat#: 1704275 |
| **Others:** | | |
| Tissue Ruptor II | Qiagen | Cat#: 9002755 |
